# Fluorescence Based on Surface Plasmon Coupled Emission for Ultrahigh Sensitivity Immunoassay of Cardiac Troponin I

**DOI:** 10.3390/biomedicines9050448

**Published:** 2021-04-21

**Authors:** Vien Thi Tran, Heongkyu Ju

**Affiliations:** 1 Department of Physics, Gachon University, Seongnam-si, Gyeonggi-do 13120, Korea; tranvien04@gmail.com; 2 Gachon Bionano Research Institute, Gachon University, Seongnam-si, Gyeonggi-do 13120, Korea

**Keywords:** fluorescence, cardiac biomarker, Troponin I, surface plasmon coupled emission, lab-on-chip optical biosensor

## Abstract

This work demonstrates the quantitative assay of cardiac Troponin I (cTnI), one of the key biomarkers for acute cardiovascular diseases (the leading cause of death worldwide) using the fluorescence-based sandwich immune reaction. Surface plasmon coupled emission (SPCE) produced by non-radiative coupling of dye molecules with surface plasmons being excitable via the reverse Kretschmann format is exploited for fluorescence-based sandwich immunoassay for quantitative detection of cTnI. The SPCE fluorescence chip utilizes the gold (2 nm)-silver (50 nm) bimetallic thin film, with which molecules of the dye Alexa 488 (conjugated with detection antibodies) make a near field coupling with the plasmonic film for SPCE. The experimental results find that the SPCE greatly improves the sensitivity via enhancing the fluorescence signal (up to 50-fold) while suppressing the photo-bleaching, permitting markedly enhanced signal-to-noise ratio. The limit of detection of 21.2 ag mL^−1^ (atto-gram mL^−1^) is obtained, the lowest ever reported to date amid those achieved by optical technologies such as luminescence and label-free optical sensing techniques. The features discovered such as ultrahigh sensitivity may prompt the presented technologies to be applied for early diagnosis of cTnI in blood, particularly for emergency medical centers overloaded with patients with acute myocardial infarction who would suffer from time-delayed diagnosis due to insufficient assay device sensitivity.

## 1. Introduction

Cardiovascular diseases, including acute coronary syndrome, have been considered the globally leading cause of human death and have been gathering great attention for their early diagnosis with cardiac markers such as myoglobin [1,2,3], C-reactive protein [4,5], B-type natriuretic peptide [6,7], cardiac Troponin T [8,9], and Troponin I (cTnI) [10,11]. Amid the biomarkers, cTnI has been conceived as one of the most important biomarkers for early diagnosing acute myocardial infarction (and stroke) due to its response sensitivity and specificity [12,13]. The cTnI concentration that remains at 1 pg mL^−1^ in serum of a normal person basically grows in response to damage to cardiac muscle and can be measured using the currently available test devices such as chemo-luminescence immunoassay and electrocardiogram.

Their limited sensitivity (~100 pg mL^−1^) of the test devices and its detection time of a couple of hours [12], however, would not allow the cTnI level ≤ 10 pg mL^−1^ in serum to be reliably measured 1–2 h after onset of the symptom such as a heart attack (whereupon its level reaches ~100 ng mL^−1^ 3–4 h after the onset) [14]. Thus, the high sensitivity assay device and the related technologies have come into a key play for early diagnosis of urgent cases of cardiovascular diseases. Extensive efforts have been made in an attempt to enhance the sensitivity in detecting cTnI concentration using the assays including electrochemical method [15,16], transistor based assay [17,18], chemiluminescence [19], fluorescence immunoassay [20], colorimetric assay [21], surface plasmon resonance (SPR) [22,23], and optical microfiber based label-free assay [10], while point-of-care testing of cTnI was reported for test device portability without compromising the sensitivity [24].

The fluorescence technique that lab-on-chip assays would adopt as the primary workhorse is known to suffer from difficulties in efficient light collection and dye photo-bleaching. These challenges, however, may be resolved by surface plasmon coupled emission (SPCE) that produces the metal enhanced fluorescence due to dye–surface plasmon near field interaction. This nano-photonics technology has been explored to amplify the fluorescence signal via applying near field plasmonic technologies to the fluorescence assay without influencing analytes [25,26]. The excited dye molecules in close proximity to a thin metal film couples with it in a non-radiative manner, generating surface plasmons at emission wavelengths (λ_em_). This near field coupling produces the directional emission at wavelengths identical to λ_em_, into an adjacent medium of a high refractive index through the metal film. The directional emission has the angles determined by the photon momentum conservation [27,28]. These angles would be identical to the incident angles of light for surface plasmon resonance (SPR) excited at λ_em_ in a Kretschmann configuration, reflecting the SPCE as the time-reversal operation of the SPR [29,30].

The SPCE that occurs via the non-radiative decay channel of excited dye molecules would increase both the fluorescence power detected and the light collection efficiency owing to the feature of directional emission. It has also been unfolded that the SPCE would reduce the photo-bleaching effects into the enhanced dye stability.

In this paper, we present the fluorescence-based sandwich immunoassay of cTnI with the SPCE technique employed for signal amplification of 50-fold at maximum. The SPCE chip fabricated includes the cTnI-specific capture antibodies on the bimetallic thin films of 2 nm-thick gold (Au)/50 nm-thick silver (Ag) layers on a glass substrate. The bimetallic SPCE based sandwich immunoassay that greatly suppresses the photo-bleaching demonstrated the ultrahigh sensitivity, with the limit of detection (LOD) of ~21.2 atto-gram (ag) mL^−1^ via the SPCE-enhanced fluorescence at the wavelength centered at 522 nm. This detection limit is lowest ever achieved to date amid those relying on optical technologies such as luminescence and label-free optical biosensor techniques. We also discuss how SPCE enhances the fluorescence signal from the sandwich assay that imposes the geometrical characteristics of molecules on the metal surface, such as the dye molecule-metal surface spacing.

The technologies presented also remove the need of using a dichroic beam splitter commonly used in a typical lab-on-chip fluorescence assay to separate emitted fluorescence from excitation source. Its simpler form potentially better suits a mini-sized assay device. The ultra-high sensitivity achievable with the SPCE chip can thus find a suitable application in biomedical assays using a simpler format of lab-on-chip based fluorescence technologies. In particular, the technologies presented for ultrahigh sensitivity assay of cTnI can open up possibility of errorless early diagnosis of cTnI in blood for effectively managing an emergency medical center, with its entry frequently overloaded with a large number of patients with acute cardiovascular diseases due to insufficient sensitivity of the current diagnostic devices.

## 2. Materials and Methods

### 2.1. Materials and Reagents

We purchased the two types of cTnI antibodies used for the sandwich immunoassay, i.e., capture antibody (clone 560, mouse) and detection antibody conjugated with the dye (Alexa fluor-488, clone 19C7, mouse) from Novus Co., (Centennial, CO, USA). Recombination Human cTnI was bought from Abcam Co., (Cambridge, UK). Cysteamine hydro-chloride (C_2_H_7_NS.HCl, 98%), *N*-hydroxysuccinimide (NHS, C_4_H_5_NO_3_, 98%), *N*-(3-dimethylaminoproyl)-*N*′-ethylcarbodiimide hydrochloride (EDC, C_8_H_17_N_3_.HCl, crystalline), (3-amininpropyl)triethoxysilance (APTES, 99%), and propanol (C_3_H_8_O, 99%) were purchased from Sigma-Aldrich Co., (St. Louis, MO, USA). Phosphate buffered saline (PBS, 1X), ultra-pure filtered water, albumin (BSA, 98%) were bought from Biosesang Co. (Seongnam-si, Korea). Pellets of Au (99.99%), Ag (99.99%) and Cr (99.99%) used for plasmonic metal coating via thermal evaporation were purchased from Taewon Scientific Co., Ltd. (iTASCO, Seoul, Korea). Polydimethylsiloxane (PDMS) was purchased from Dow Corning Co., (Midland, MI, USA).

### 2.2. Plasmonic Chip Fabrication

**Figure 1:**
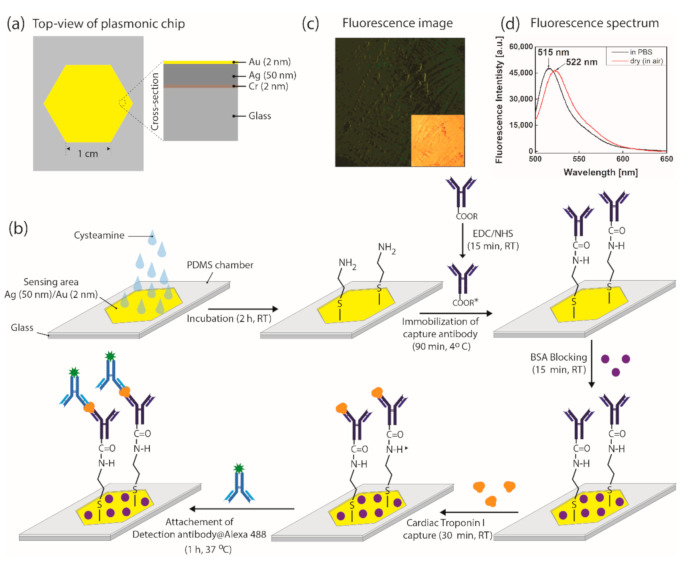
(**a**) Top-view and cross-section of the SPCE chip. (**b**) Procedures for the sandwich immune reaction for detecting cTnI. (**c**) Fluorescence microscope image of the green color due to fluorescence from the SPCE chip that went through the procedures of Figure 1b, under excitation (blue) light illumination (inset shows the image under white light illumination). (**d**) The fluorescence spectrum of Alexa 488 conjugated D-Ab of 1 µg mL^−1^ in PBS solution and in air under illumination of LED at 470 nm wavelength.

We made the PDMS structure, which has a thick-walled hollow inner space with its open top looking like an equilateral hexagon (side length of 1 cm) as shown in Figure 1a (top view). We used an oxygen plasma to bond it to a soda lime glass substrate (Marienfield, Germany), generating a chamber of a hexagonal pillar shape with its volume of 200 µL. A thermal evaporator (DAEKI HI-TECH Co., Ltd., Daejeon, Korea) was used to coat the film layers of 2 nm-thick chromium (Cr), 50 nm-thick Ag and 2 nm-thick Au consecutively on glass bottom of the PDMS walled chamber at the deposition speed of 0.5 Å s^−1^ for both Ag and Au, and 0.1 Å s^−1^ for Cr (monitored by a quartz crystal microbalance (QCM, QI8010, Fil-Tech Inc., Boston, MA, USA)), at the pressure ≤ 7 × 10^−6^ Torr. 

The Cr layer acts as an adhesive layer for coating the Ag layer on a glass substrate [31]. The 2 nm-thick Au layer enables the thiol-linking to be used for immobilizing the capture probes, while acting as anti-oxidization protective layer for the inner Ag layer [26,32]. Use of the Ag layer as the prime metallic film for surface plasmon is supported by its higher quality factor, indicating a greater number of dye molecules coupled for more highly effective SPCE than in a case of an Au layer at visible wavelengths [26,32,33,34,35,36].

Care has to be taken to ensure that the illumination area by the dye excitation light source did not exceed the hexagonal area of the chamber bottom to avoid unwanted scattering of light with chamber wall.

### 2.3. Procedures for the Sandwich Immune Reaction on the Surface of SPCE Chip

Figure 1b shows the procedures of the sandwich immunoassay of cTnI on the SPCE chip. Its outermost surface of Au film is modified using cysteamine of 10 mM (diluted in deionized water) for 2 h at room temperature (RT), to generate amine group on it. The cTnI capture antibody (C-Ab) of 2 µg mL^−1^ concentration is introduced to the amine-modified surface. The C-Ab binding amine group is taken in 90 min at 4 °C. Prior to immobilizing the C-Ab, we activate its carboxylic groups in 15 min by immersing it in the solution of 1,4-dioxane, toluene, 1-ethyl-3-(3-(dimethylamino)propyl) carbodiimide (EDC) and *N*-hydroxysuccinimide (NHS) with phosphate buffered saline (PBS) solvent. The bovine serum albumin (BSA) of 2 mg mL^−1^ is in turn injected to the surface and incubated in 15min as the blocking agents to avoid non-specific binding of cTnI with the Au surface. Then the target molecules and cTnI of various concentrations are injected to the C-Ab modified surface in 30 min. Next, the D-Ab@Alexa 488 of 1 µg mL^−1^ is injected in 1 h at 37 °C to complete the sandwich immune reaction, with the chip surface being ready for the SPCE-enhanced fluorescence. 

It is noted that the concentrations of the C-Ab and D-Ab@Alexa 488 all need to exceed that of cTnI injected to avoid unwanted saturation of cTnI assayed. All bio-agents are diluted in PBS solvent. The surface is rinsed with PBS solvent immediately after every immobilization procedure mentioned above. Finally, the SPCE chip is dried in an oven at 37 °C for 30 min to make an air ambient that determines the SPCE directional angle, before being installed in the optical setup for fluorescence detection.

We also prepare the control chip to detect the fluorescence without SPCE enhancement, reflecting the fluorescence signal similar to that obtainable by a conventional fluorescence technique. Its signal magnitude is used as a reference to estimate the amplification factor of the SPCE-enhanced fluorescence. The control chip is designed to have the same layers as the SPCE chip, i.e., C-Ab with blocking agent, cTnI, and D-Ab@Alexa 488, except those of the bimetallic film. Therefore, for control chip fabrication, the slide glass surface undergoes the oxygen plasma treatment prior to being incubated in the APTES (volume concentration of 2% in propanol) to generate the amine-modified surface. We use the procedures for immobilizing, on the control chip, C-Ab, BSA (blocking agents), cTnI, and D-Ab@Alexa 488, exactly in the same ways as those mentioned above for the SPCE chip.

As shown in Figure 1c, we make a visual check of the fluorescence from the dye on surface of the SPCE chip (that undergoes the procedures in Figure 1b), using a fluorescence microscope (Eclipse 80i, Nikon, Japan) designed to detect the fluorescence emitting into air (cTnI concentration of 6.6 µg mL^−1^). Under illumination of blue excitation light, green fluorescence is clearly visible while its inset shows an image under white light illumination. Observation of the green fluorescence verifies the successful immobilization of D-Ab@Alexa 488 through the sandwich immune reaction described above.

Figure 1d shows the spectra of fluorescence from D-Ab@Alexa 488 in dried condition, measured by a spectrometer (Ocean Optics USB 2000+, Orlando, FL, USA). The spectrum peaks at 515 nm when the D-Ab@Alexa 488 is in the PBS solvent. The peak shifts to 522 nm in dried condition [37]. Such a shift is taken into account to choose the pass band filter that allows only fluorescence light at λ_em_ while blocking light at λ_ex_ for detection. It is worth noting that the extremely low levels of the cTnI concentrations ranging from 0.01 pg mL^−1^ to 0.5 pg mL^−1^ (PBS buffer) cannot serve for the visual check of the dye green color when using the fluorescence microscope. It is noted that, for a given excitation power, at each concentration (including zero concentration), three independent measurements are conducted to obtain an error bar. Each measurement includes recording the signal for 10 s with the data sampling frequency of 1 Hz.

### 2.4. Instrument and Apparatus for Detecting SPCE-Enhanced Fluorescence

**Figure 2:**
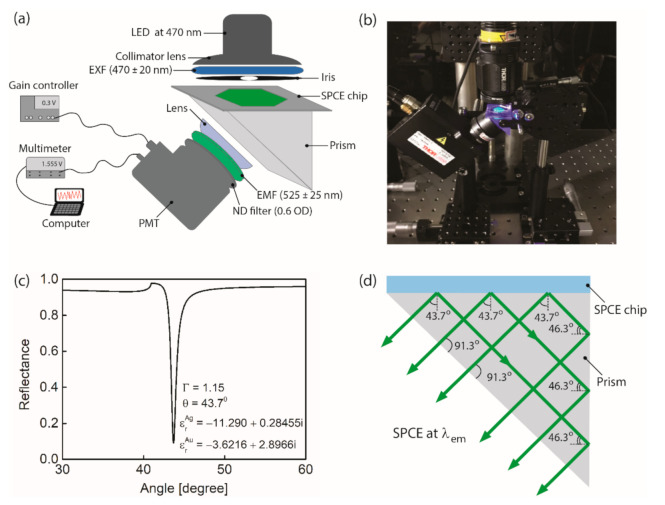
(**a**) Schematic of the experimental setup (ND filter: neutral density filter, OD: optical density) and (**b**) a photo of the setup. (**c**) Calculated reflectance versus incident angle in the N-BK7 prism based Kretschmann configuration for SPR excitation at λ_em_ ($\varepsilon_{r}^{\mathrm{Ag}}$ and $\varepsilon_{r}^{\mathrm{Au}}$: relative permittivity for Ag and Au [38,39], Γ: the depth-to-width ratio of the dip). (**d**) Prism-aided redirection of the SPCE directional rays of light.

Figure 2a,b shows schematic and the photo of the setup for detecting the SPCE-enhanced fluorescence. The fluorescence excitation source is the light emitting diode (LED) with its central wavelength of 470 nm (M470L3, Thorlabs Inc., Newton, MA, USA), its output power being controllable via a current supplier. The source light passes through the excitation filter (EXF) (470 nm ± 20 nm, Chroma Co., Bellows Falls, VT, USA) into a circular cross-sectional beam of 1 cm diameter. The adjustable iris with which we follow the EXF could control the illumination area on surface of the SPCE chip. The chip is installed on one side surface of a right-angled prism of N-BK7. Index-matching oil (MOIL-10LF, Leica Type F, Thorlabs Inc., Newton, MA, USA) is inserted to fill the gap between the glass bottom of the chip and the prism surface to avoid unwanted total internal reflection induced by index discontinuity.

Figure 2c shows the computed optical reflectance versus incident angle of light in the N-BK7 prism-based Kretschmann configuration with the bimetallic film, i.e., 2 nm thick Au/50 nm thick Ag layers, assumed for SPR excitation at λ_em_ (not λ_ex_). The incident angle at the reflectance minimum being calculated as 43.7° is considered the same as the angle of the SPCE light at λ_em_ [40]. The depth-to-width ratio of the reflection dip, i.e., the Γ which is in proportion to the plasmonic resonance quality factor, turns out to be comparable to that of the single Ag film with the thickness optimized. This bimetallic film nearly optimizes the efficiency of the dye–metal film coupling into the SPCE while providing the Au surface flexibility for biochemical treatment required for the sandwich immune reaction. Figure 2d depicts the prism-based redirection of the SPCE induced directional rays of light on a given triangular cross section. The ray–surface angles at which light exits the prism base surface reaches ~90° (nearly zero incident angles), permitting nearly full transmission through air–prism boundary with negligible internal reflection.

The light collection optics comprises a plano-convex lens of a 30 mm focal length, the emission filter (EMF) (Chroma Co., Bellows Falls, VT, USA) with its pass band of 525 ± 25 nm, and a neutral density (ND) filter. The collected light is then detected by a photomultiplier tube (PMT) (PMM02, Thorlabs Inc., Newton, MA, USA) connected with a multi-meter (Tektronix DMM 4050, 6-1/2 Digit Precision Multimeter, Beaverton, OR, USA) which is interfaced with a data acquisition computer. We set the PMT gain voltage as 0.3 V and the ND filter optical density (OD) as 0.6, both to avoid signal saturation and to enable noise floor to be detectable as the minimum possible value. We then keep these parameters fixed during measurement for all concentrations of cTnI used.

It should, however, be mentioned that the fact that the SPCE produces the 3-dimensional conical direction of light rays must generate the complex internal reflections, given the asymmetrical inner structure of the prism, such as reflections azimuthally slanted and skew from those shown in Figure 2d. This would incur the far-from-zero incident angles of rays at the prism base–air interface, leading to inevitable loss of light for detection in the present setup.

## 3. Results and Discussion

**Figure 3:**
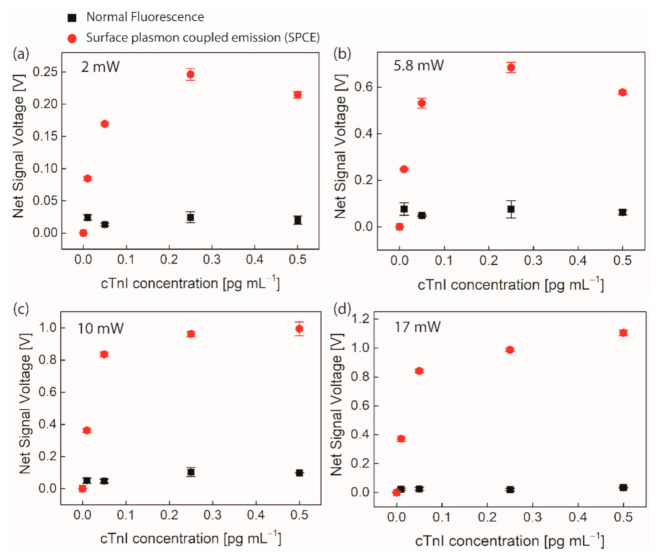
The net fluorescence signal versus cTnI concentration under various incident powers of excitation source (**a**) 2 mW, (**b**) 5.8 mW, (**c**) 10 mW, and (**d**) 17 mW. Error bar = ±SD and *n* = 3.

Figure 3a–d show the net fluorescence signal versus cTnI concentrations, under various optical powers of excitation light incident to the chip, i.e., 2, 5.8, 10, and 17 mW. The net signal (vertical axis) is obtained by subtracting the signal measured without cTnI, namely, the background noise from the one measured with cTnI, eliminating effects of auto-fluorescence and unwanted leak of excitation light through a spectral filter into a detector. Red solid circles represent the fluorescence signal detected through the prism based SPCE chip, while black solid squares represent the reference fluorescence signal detected in the same manner using the control chip at each cTnI concentration. This allows us to compare the net fluorescence signals obtained between two techniques, i.e., the presented SPCE technology and a conventional fluorescence one. 

It is found that use of the control chips hardly distinguish the signals from the different concentrations of cTnI while use of the SPCE chips do. This is primarily due to the photo-bleaching and the saturation effects of the excited dye molecules on the control chip, which began to be significant already at the low concentrations used. 

In contrast, the SPCE chip produces much higher signal-to-noise (S/N) ratio than the control chip at each concentration of cTnI for all incident excitation powers. This enables the signals from different concentrations to be markedly distinguishable, especially at the low concentration range as shown in Figure 3a–d. The LOD is defined as the concentration that produces the signal corresponding to three times the standard deviation at zero concentration. It is estimated as 22.2, 21.7, 21.2, and 26.5 ag mL^−1^ for excitation powers of 2, 5.8, 10, and 17 mW with coefficient of variation (CV) < 5.2%, respectively. For LOD estimation, we use linear regression of the data at small concentration close to zero, being considered a good approximation as seen in Figure 3a–d. These levels of LODs presented are recognized as the lowest ever reported to date amid those obtained using optical technologies, such as label-free optical sensors and luminescence [10,19,22,41,42,43,44,45] as shown in Table 1, where all LODs were reported using PBS without human serum.

It is worth noting that the SPCE technologies not only enhance the fluorescent signals detected but also suppress photobleaching due to the near field coupling of excited state dye molecules with thin metal layers that support surface plasmon resonance. To comprehend the superior performance of the SPCE chip over the control chip, let us begin by considering that the LED light source excites the molecules of the dye, the Alexa 488, which are conjugated with the detection antibody (D-Ab@Alexa 488) for the sandwich immune reaction. The excited states of dye molecules make a near-field coupling with the bimetallic film for surface plasmons at λ_em_. The non-radiative coupling of the excited dye molecules into surface plasmons at the dielectric–metal interface, a sort of non-radiative decay of a dye molecule, occurs only for those spaced close to the metal film, i.e., those within the evanescent field depth from the outermost metal (Au) surface of the SPCE chip. This non-radiative decay rate (γ_sp_) that can be boosted by the enhanced density of local photonic modes of surface plasmons [46,47] can contribute to the total decay rates (γ_t_) of a dye emitter as given by

(1) $$\gamma_{t}=\gamma_{\mathrm{se}}+\gamma_{\mathrm{sp}}+\gamma_{\mathrm{nr}}$$

where the γ_se_ is the radiative decay rate due to the spontaneous emission while we group the other possible non-radiative decay processes with the single rate constant γ_nr_. Eventually, the presence of non-radiative decay channel, γ_sp_, would produce the directional emission of light at a specific angle into the prism (see Figure 2d) at the same wavelength as the dye fluorescent emission as is termed SPCE. The prism-based light collection would harvest the benefits of the directional emission for light collection efficiency. Therefore, the favorable contribution of the SPCE with its directional properties to the fluorescence detection would increase the S/N ratio.

Moreover, this non-radiative decay channel driven by $\gamma_{\mathrm{sp}}$ decreases the probability of coupling between excited dye molecules themselves, leading to photobleaching suppression. As demonstrated in Figure 3a–d, the SPCE chip reduces photobleaching significantly while the control chips severely suffer it with increasing the concentration.

It is interesting to note that, though signal suffers from severe saturation, it still rises with concentration increasing to the highest one (0.5 pg mL^−1^) only for higher excitation powers used. It is not clear for this behavior but the presence of elastic scattering of excitation light with cTnI/D-Ab@Alexa 488, which can in turn leak through the emission filter, may be one of the reasons. This noise cannot be ruled out by the process of subtracting the background noise from the measured signal. 

We here define the enhancement factor (η) of the detected fluorescence as given by

(2) $$\eta \equiv S_{\mathrm{SPCE}}/S_{\mathrm{control}}$$

where the S_SPCE_ is the net signal (voltage) obtained with the SPCE-chip while the S_control_ is that obtained with the control chip at each concentration as seen in Figure 3a–d. The bimetallic SPCE immunoassay technologies permit us to achieve the significant enhancement under each incident excitation power as shown in Figure S1 (Section 1 of Supplementary Materials). We achieved the maximum η of ~50-fold when using the highest incident power that produces the largest number of excited dye molecules that can, in turn, possibly lead to the largest number of the SPCE-generated photons. 

The fitting curves to the measured data for quantitative detection of cTnI are also shown in Figure S2. The function used for calibration purpose fitting describes the signal saturation as significantly weakened compared to the control case. The net fluorescence signal still gets higher at higher concentrations, though its rate reduces with increasing cTnI concentration due to the non-vanishing effects of photobleaching.

In these SPCE technologies, excitation photons that do not excite dye molecules are not allowed to directly couple into the prism across the metallic layers due largely to the metal induced light attenuation (~87%). This attenuation effect can eliminate the need of using optical component that separates the excitation light from fluorescence signal, such as a dichroic beam splitter commonly found in a typical fluorescence-based lab-on-chip to suppress the noise power at λ_ex_. The quasi-straight geometry of optical components aligned for detecting SPCE (with no beam splitter) from an excitation light source to a detector (see Figure 2a)) would facilitate packaging into the lab-on-chip setup of a simpler form than the typical one.

It is also worth noting that the SPCE quenching would occur for the dye molecules spaced less than 10 nm from the metal surface due to the non-radiative energy transfer to the metal similar to the Förster resonance energy transfer (FRET) [29]. The layers of the cTnI capture antibody (C-Ab) and the analyte (cTnI), however, would act as the spacer layers to reduce such quenching effects in this SPCE fluorescence based on sandwich immune reaction.

Meanwhile, the fact that the excited dye molecules cannot produce the s-polarized SPCE limits the further enhancement of S/N ratio in fluorescence detection. The waveguide layer containing the dye-molecules, instead, might be added on the bimetallic film for the s-polarized SPCE coupling into the direction emission [48], though it would not be straightforward to incorporate the dye and the protein elements comprising the sandwich immune reaction into the waveguide. 

The time taken for procedures of sandwich reaction after having C-Ab immobilized on the surface, e.g., the bond between C-Ab and cTnI (~30 min), and that between cTnI and D-Ab@Alexa 488 (~60 min) is ~90 min. This leads to the possibility that, given the dynamic antibodies developed to shorten the immune reaction time, the ultrahigh sensitivity available by the presented SPCE technologies would make it plausible to diagnose the cTnI level in blood in amuch earlier stage.

It is also worthwhile to note that the LOD, however, may be likely to be worse than the current one when using human sera that contain various kinds of proteins. This is because they can induce unwanted non-specific binding (false signal) that would increase the background signal noise. Use of surface rinsing and blocking medium included in the surface treatment of the present work may reduce such unwanted noise and thus prevent the very high sensitivity demonstrated in the present device from being tarnished. 

## 4. Conclusions

This work demonstrates the SPCE based fluorescence detection for quantitative assay of cTnI using the sandwich immune reaction. With greatly suppressed photo-bleaching, remarkably enhanced fluorescence is detected with the enhancement factor of up to 50-fold. The dye–bimetallic film near-field coupling produces the SPCE induced directional emission at λ_em_, collected by a prism without a dichroic beam splitter commonly used in a typical lab-on-chip fluorescence device. Signal fluctuations of zero concentration turns out to be much smaller than the differential change that occurs as concentration rises to the minimum concentration used. This leads us to estimate the detection limit of 21.2 ag mL^−1^ being the lowest ever reported to date amid those obtained by optical technologies, such as label-free optical bio-sensing and luminescence-based assay where cTnI in PBS buffer is used (not in human sera).

It is also noted that the fact that the near field coupling cannot occur for the s-polarized SPCE limits further enhancement in detecting fluorescence. However, further enhancement may be accomplished by adding a waveguide layer on top of the bimetallic film to simultaneously obtain the s-polarized directional emission in addition to the p-polarized one for even higher sensitivity [27,48,49,50]. In this case, dipoles of the fluorescent dyes oriented in parallel to the s-polarization can also be coupled with waveguide modes, subsequently producing s-polarized direction emission at an angle different from the angle of SPCE directional emission. This may additionally benefit early diagnosis of cTnI to shorten the whole sandwich immune reaction via using dynamic antibodies that can bond with cTnI within a much shorter reaction time while approximately maintaining the bio-affinity and bond strength. 

This work presented does not use human sera that would contain many other kinds of proteins, while focusing on adaptability of the SPCE technologies to cTnI sandwich assay using pure cTnI in PBS buffer. The protocol of the sensing surface treatment that includes the sandwich immunoassay with DI water rinsing and use of blocking solution, however, would be able to detect cTnI specifically together with minimizing non-specific adsorption under presence of other substances such as in human sera. This can strengthen utility of the ultrahigh sensitivity of the sensor transduction demonstrated in the present work.

Future works shall cover the clinical application of the presented SPCE technologies for cTnI detection in human sera, expecting the LOD to be about an order-of-magnitude higher than the atto-g mL^−1^ level at worst. However, this level still would be very low (still good sensitivity), deserving further investigation with the surface treatment to remove unwanted non-specific bonding. In addition, use of dynamic antibodies may shorten the detection time considerably, being more favorable for early diagnosis of cTnI.

The technologies presented for the ultrahigh sensitivity of cTnI assay can also potentially open up an opportunity of removing the need that patients with emergent cardiovascular diseases, such as acute coronary syndromes, should wait several hours until their level in blood rises to the level detectable by the assay devices and kits currently available in the emergency medical center.

## Figures and Tables

**Table 1:** The comparison of this work and previous report for cTnI detection using optical technologies.

cTnI Detection Methods	Concentration Range	LOD (with No Serum Used)	Ref.
Surface plasmon coupled emission (SPCE)	0–0.5 pg mL^−1^	21.1 ag mL^−1^	Present work
Optical microfiber coupler	2–10 fg mL^−1^	2 fg mL^−1^	[10]
Chemiluminescence	0–1.5 ng mL^−1^	0.012 ng mL^−1^	[19]
Electrochemiluminescence	1 fg mL^−1^–1 μg mL^−1^	0.58 fg mL^−1^	[41]
Photoelectrochemistry	0.0005–1000 ng mL^−1^	0.18 pg mL^−1^	[42]
Fluorescence	0.05–32 ng mL^−1^	0.032 ng mL^−1^	[43]
Magnetic field-assisted Surface plasmon resonance	50–125 μg mL^−1^	1.25 ng mL^−1^	[44]
Surface plasmon resonance	0–0.160 μg mL^−1^	0.068 ng mL^−1^	[22]
Optical solution immersed silicon	0.005–10 ng mL^−1^	5 and 10 pg mL^−1^	[45]

## Data Availability

The data presented in this study are available on request from the corresponding author. The data are not publicly available due to privacy.

## Supplementary Materials

The following are available online at https://www.mdpi.com/article/10.3390/biomedicines9050448/s1, Figure S1: The enhancement factor (η) of the detected fluorescence, Figure S2: The curve fitting function of measured data for quantitative detection of cTnI.
