# Supplementary material for: Fluorescence Based on Surface Plasmon Coupled Emission for Ultrahigh Sensitivity Immunoassay of Cardiac Troponin I
Source: Biomedicines. 2021 Apr 21;9(5):448. doi: 10.3390/biomedicines9050448 (PMC8143139; doi:10.3390/biomedicines9050448)
Supplement: Supplementary file 1 [file biomedicines-09-00448-s001.zip › biomedicines-1168938-SI.pdf]

biomedicines

MDPI

Supplementary Materials

Section 1. The enhancement factor (η) of the detected fluorescence

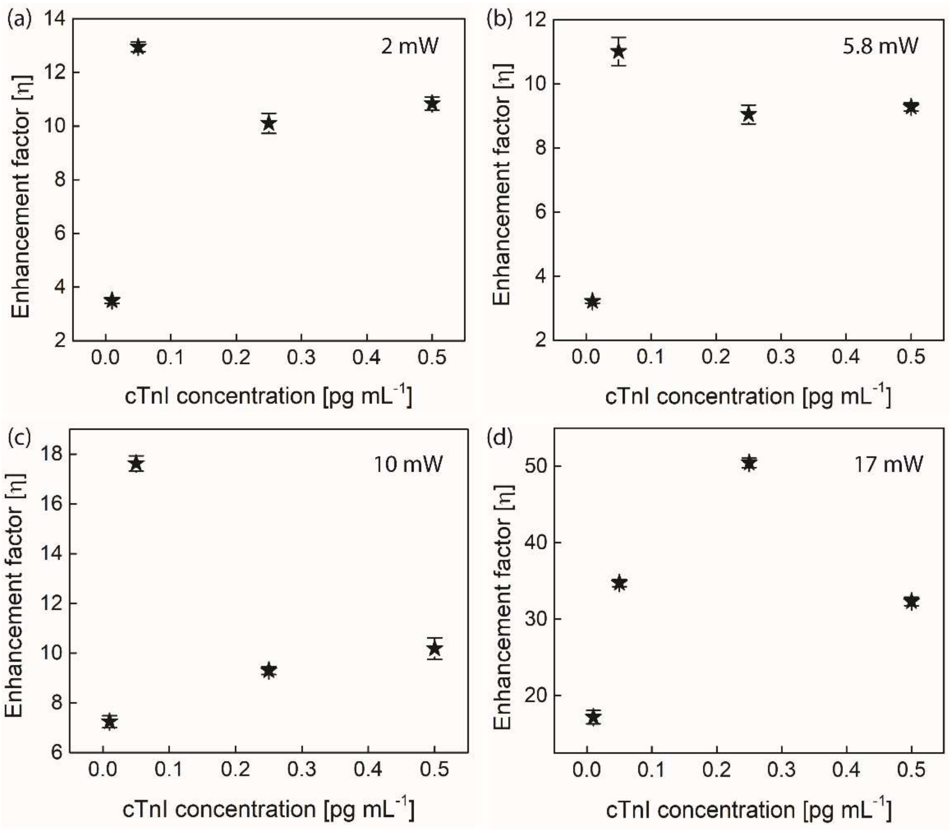

Figure S1. The enhancement factor (η) of the detected fluoresence signal versus cTnI concentration for different excitaiton powers of (**a**) 2 mW, (**b**) 5.8 mW, (**c**) 10 mW and (**d**) 17 mW.

Section 2. The curve fitting function of measured data for quantitative detection of cTnI

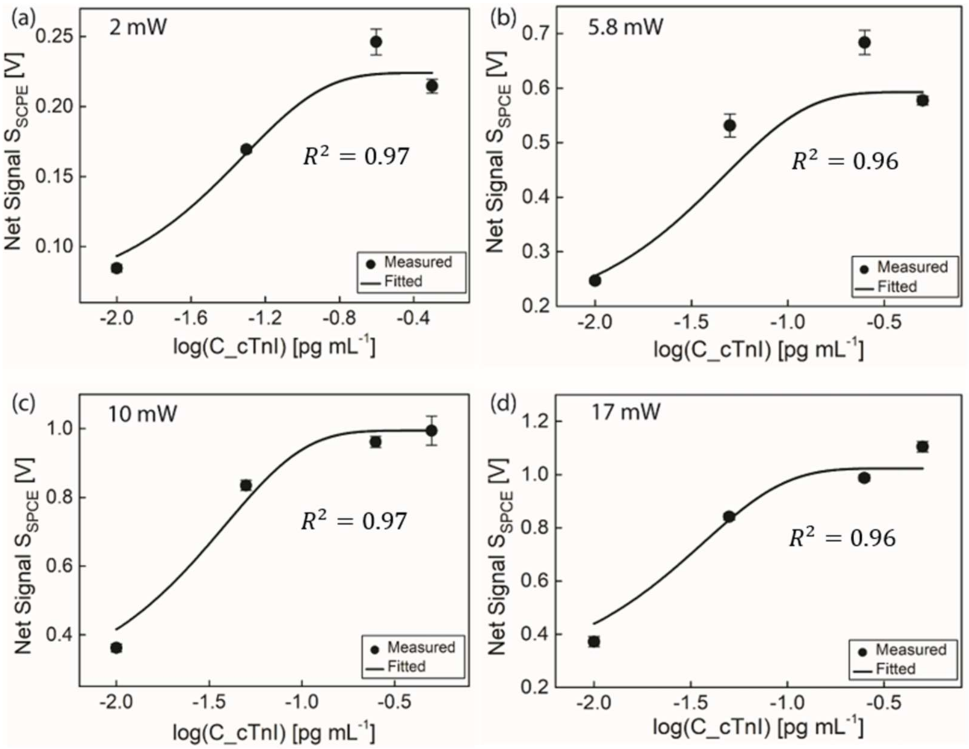

Figure S2. Fitting the function of the form $S_{SPCE} = S_0 + f_1[1 - \exp(-f_2 C_{cTnI})]$ to the measured data for quantitative detection of cTnI concentration, C_{cTnI}. The f_1 and f_2 are the fitting parameters while S_0 is obtained by estimating the linear slope at $C_{cTnI} = 0$ extended from the low concentration data. (a) $S_0 = 0.063,\ f_1 = 0.16,\ f_2 = -20.6$ for excitation power of 2 mW, (b) $S_0 = 0.17,\ f_1 = 0.42,\ f_2 = 21.3$ for 5.8 mW, (c) $S_0 = 0.24,\ f_1 = 0,\ f_2 = 26.0$ for 10mW, (d) $S_0 = 0.25,\ f_1 = 0.77,\ f_2 = 27.5$ for 17 mW.
